# Dr. Sabar Mirza Farman Farmaian; Benefactor and Former Director of Pasteur Institute of Iran

**Published:** 2018-01

**Authors:** Narges Shahbazi, Ehsan Mostafavi

**Affiliations:** Department of Epidemiology and Biostatistics, Research Centre for Emerging and Reemerging Infectious Diseases, Pasteur Institute of Iran, Tehran, Iran. E-mail: mostafaviehsan@gmail.com


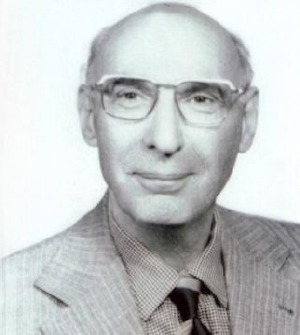


Pasteur Institute of Iran (PII) is known for its history of benefaction by exquisite characters, the most prominent of who is the family of Farman Farmaian.

Dr. Sabar Mirza Farman Farmaian, born in 1912 in Tehran, resided as the director of PII for a period of six years (1971-1977). Furthermore, he devoted his house (located in Shemiranat) for the establishment of a center to study and combat infectious diseases. Both of these events had a significant impact on the fate of PII.

He was born to a famous family of Farman Farmaian. His father, Abdol-Hossein Mirza Farman Farmaian, the grandson of Abbas Mirza and Fath-Ali Shah, was born in 1852, in Tabriz. He was known as “Salar Lashkar” and “Farman Farma”. He finished his elementary studies at Dar ul-Funun, after which he went to an Austrian school to learn military skills. He held numerous critical positions during 1881-1919. These include the chief of Kerman and Azerbaijan military troops, governor of Kerman, Tehran, Fars, Khorasan, and Kermanshah, as well as the minister of War, Justice and the Interior. The most prominent of all is his chair as the prime minister during the reign of Ahmad Shah Qajar.

**Fig. 1:**
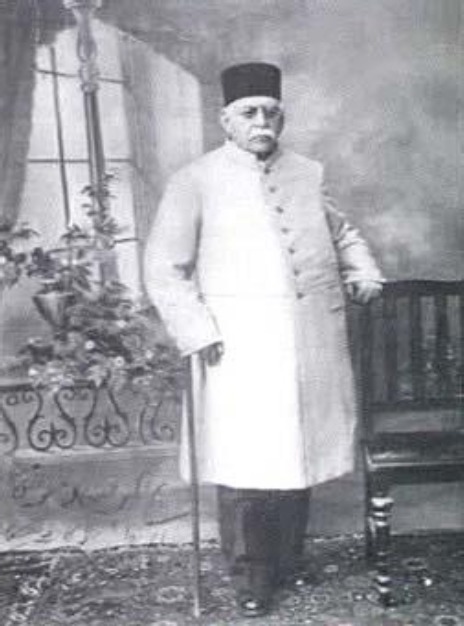
Abdol-Hossein Mirza Farman Farmaian (1852-1939), father of Dr. Mirza Farman Farmaian and one of the most prominent Qajar princes.

In 1922, due to his passion for promotion of health, Abdol-Hossein Farman Farmaian dedicated a vast piece of land (~13 thousand square meters with the value of 10 thousand tomans, at the time) for the expansion of Pasteur Institute of Iran. He is also the benefactor of other medical establishments, including the well-known Firoozabadi Hospital, in Tehran. His five sons, namely Firouz Nosrat-ed-Dowleh, Sabar Mirza, Abbas Mirza, Mohammad Vali Mirza, and Mohammad Hossein Mirza Firouz, all held ministerial positions at one time or another[1,2].

Initially, Firouz Nosrat-ed-Dowleh, who was the Foreign Minister of Ahmad Shah Qajar in 1919, in cooperation with Zka’almlk, Dr. Mohammad Loqman-e-Adham, Dr. Hakim al- Doleh and Dr. Mahmoud Khan, established Pasteur Institute of Iran, based on the Paris Memorandum of Understanding.

Sabar Mirza Farman Farmaian was trained at home in childhood. Sabar Mirza was a small boy who went to Fars Province for one of the missions of his father who was the governor of this province. At the end of the mission, the eight-year-old Sabar Mirza returned to Tehran. At the age of 12, Sabar went to France to continue his education by his father request. After receiving diploma, he started to study medicine in France. He continued his study in medicine in Switzerland and received his degree in 1983 from the University of Geneva[2].

**Fig. 2:**
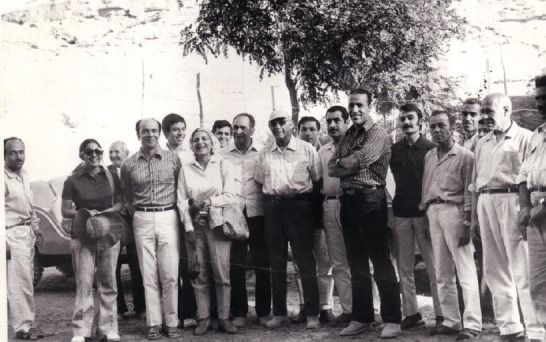
WHO meeting on plague, the research centre of emerging and reemerging infectious diseases, the branch of Pasteur Institute of Iran in Akanlu, Hamadan, August 1975; Dr. Sabar Farman Farmaian is with a hat in the middle.

In 1939, due to his father’s illness, he returned to Iran. After his father’s death, he spent his two-year military service as the physician in the army. Then, in the ministry of health, he was the supervisor of health problems in the west of the country. In the summer of 1942, he was appointed as the head of the health center in Hamadan and continued his job until 1943. He was then appointed as the manager of the Malaria Eradication and Control Program in Iran. He was able to control this disease extensively in Iran in three years.

Due to his interest, he went to England to study communicable and tropical diseases and received his public health diploma from London School of Hygiene and Tropical Medicine in 1946. He took care of his younger brother and sisters and did his best for the well-being of Farman Farmaian Family[2].

Dr. Farman Farmaian was one of the strong supporters of Dr. Mohammad Mossadegh during the nationalization of the Iran oil industry movement in 1953. On the 26^th^ July 1952, he became the minister of health in Mossadegh government and continued till 8th June 1953. Then, for a short time, he became the governor of Fars Province. After 1953 Coup and Dr. Mossadegh’s fall, Sabar was forced to leave Iran. Immediately, the World Health Organization (WHO) invited him to work as its representative in Beirut and Cairo during which he implemented various health projects in African countries. He was selected as an expert in the scientific committees of WHO.

In 1960, Dr. Sabar attended the 13^th^ WHO Malaria expert meeting in Geneva, as the head of the Department of Tropical Medicine of Tehran University and head of the Institute of Parasitology and Malariology. There, he was selected as one of the main members of the committee for controlling Malaria in the south-west of Asia, including Philippines, Cambodia and Vietnam. He also worked as WHO advisor for some time. In this regard, WHO appointed him as one of the best Malaria experts known in the world.

In 1971, the Iranian government invited Dr. Sabar to come back to Iran and work as the director of Pasteur Institute of Iran. He was in this position for six years (1971-1977) before being retired. At that time, production and research in Pasteur institute of Iran were conducted in a single building. Dr. Sabar decided to separate them. Therefore, he purchased a land at Karaj Road for this purpose. He also provided modern laboratory devices for the institute. Employees of PII remember Dr. Sabar’s time as a time of good management, good behavior and effort[3,4].

After being retired, he continued medicine at his home in Shemiranat. Farman Farmaian Family started a fellowship program for the researchers who were interested in the social, political history, culture and civilization of Iran with focus on the Qajar dynasty (1794-1925) in the International Institute of Social History in Amsterdam, the Netherlands.

**Fig. 3:**
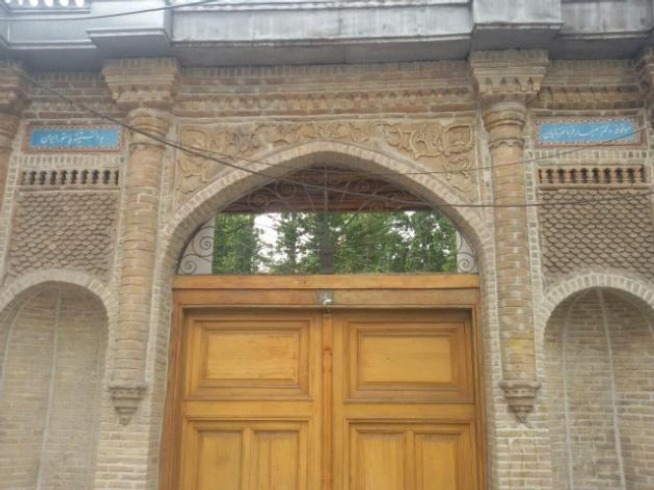
New branch of Pasteur Institute of Iran in Shemiranat devoted by Dr. Sabar Farman Farmaian in 2005.

Few months before his death, on November 2005, Dr. Sabar devoted his home in Shemiranat (4843 square meters, including a garden and four stores) for the establishment of a vaccination clinic against infectious diseases as well as specialized laboratories of Pasteur Institute of Iran for medical researchers. This new branch of PII started to work on October 2007. This place, formerly, was used as the summer camp of Abdol-Hossein Mirza Farman Farmaian when, in 1929, it was given to Sabar Mirza Farman Farmaian. In addition, proceeds from the sale of his furniture were all spent on studying medical students. Dr. Farman Farmaian died on May 19th, 2006. He was buried in Behesht-e Zahra Cemetery in Tehran. He never got married.

## Lessons from Dr. Sabar Farman Farmaian’s Life

One of the reasons of Dr. Sabar’s success was his growth in a big and rich family who was interested in science and could dispatch their children to aboard for continuing their education. Dr. Sabar brother, Firouz Nosrat-ed-Dowleh, was one of the first creators of the Pasteur Institute of Iran and his father was the devotee of this institute. For six years, he was the director of the Pasteur Institute of Iran and devoted his home for improving the institute. Farman Farmaian Family and the Pasteur Institute of Iran are closely related to each other. Owing to the feeling of other-kindness, he devoted part of his life to control malaria in different parts of the world, including Asia and Africa. He was a warm-blooded man working with great people like Dr. Mohammad Mossadegh, his cousin. His valuable life is a worthy part of history of medicine in Iran.

Peace be upon his soul.

## References

[1] Azizi MH, Nayernouri T (2008). The establishment and the first four decades of the activities of the Pasteur Institute of Iran. Archives of Iranian medicine.

[2] Farmaian F, Munke F, Munke D (1993). Daughter of Persia:a woman's journey from her father's harem through the Islamic Revolution.

[3] Ghodsi M, Mostafavi E (2016). Memories of school days and years of service at the Pasteur Institute of Iran.

[4] Mainbourg J, Yousefi Behzadi M, Mostafavi E (2015). Marcel Baltazard:Adventure of Plague.

